# How useful is the EQ-5D in assessing the impact of caring for people with Alzheimer’s disease?

**DOI:** 10.1186/s12955-017-0591-2

**Published:** 2017-01-21

**Authors:** Catherine Reed, Annabel Barrett, Jeremie Lebrec, Richard Dodel, Roy W. Jones, Bruno Vellas, Anders Wimo, Josep Maria Argimon, Giuseppe Bruno, Josep Maria Haro

**Affiliations:** 1 Eli Lilly and Company Limited, Erl Wood Manor, Sunninghill Road, Windlesham, Surrey GU20 6PH UK; 2 Lilly Deutschland GmbH, Bad Homburg, Germany; 3 Philipps-University, Marburg, Germany; 4 RICE (The Research Institute for the Care of Older People), Royal United Hospital, Bath, UK; 5 Toulouse University Hospital, Toulouse, France; 6 Karolinska Institute, Stockholm, Sweden; 7 Servei Català de la Salut, Barcelona, Spain; 8 Sapienza University of Rome, Rome, Italy; 9 Parc Sanitari Sant Joan de Déu, CIBERSAM, Universitat de Barcelona, Barcelona, Spain

**Keywords:** Alzheimer’s disease, Caregiver burden, Europe, Health-related quality of life, Informal care, Observational study

## Abstract

**Background:**

The impact on informal caregivers of caring for people with Alzheimer's disease (AD) dementia can be substantial, but it remains unclear which measures(s) best assess such impact. Our objective was to use data from the GERAS study to assess the ability of the EuroQol 5-dimension questionnaire (EQ-5D) to measure the impact on caregivers of caring for people with AD dementia and to examine correlations between EQ-5D and caregiver burden.

**Methods:**

GERAS was a prospective, non-interventional cohort study in community-dwelling patients with AD dementia and their informal caregivers. The EQ-5D and Zarit Burden Interview (ZBI) were used to measure health-related quality of life and caregiver burden, respectively. Resource-use data collected included caregiver time spent with the patient on activities of daily living (ADL). Spearman correlations were computed between EQ-5D scores, ZBI scores, and time spent on instrumental ADL (T-IADL) at baseline, 18 months, and for 18-month change scores. T-IADL and ZBI change scores were summarized by EQ-5D domain change category (better/stable/worse).

**Results:**

At baseline, 1495 caregivers had mean EQ-5D index scores of 0.86, 0.85, and 0.82, and ZBI total scores of 24.6, 29.4, and 34.1 for patients with mild, moderate, and moderately severe/severe AD dementia, respectively. Change in T-IADL showed a stronger correlation with change in ZBI (0.12; *P* < 0.001) than with change in EQ-5D index score (0.02; *P* = 0.546) although both correlations were very weak. Worsening within EQ-5D domains was associated with increases in ZBI scores, although 68%–90% of caregivers remained stable within each EQ-5D domain. There was no clear pattern for change in T-IADL by change in EQ-5D domain.

**Conclusions:**

EQ-5D may not be the optimum measure of the impact of caring for people with AD dementia due to its focus on physical health. Alternative measures need further investigation.

## Background

The impact of caring for people with Alzheimer’s disease (AD) dementia falls heavily on informal caregivers who are increasingly required to assist with activities of daily living (ADL) and make decisions on behalf of patients as the disease progresses. The health impact on caregivers can be both physical and emotional [1]; nearly half of the caregivers in some studies meet formal diagnostic criteria for depression and show increased use of health services and psychiatric medication [2]. Caring for a person with AD dementia can also have a social and financial impact, and the overall effect on caregivers is thought to be reflected in deterioration in their health-related quality of life (HRQoL) as patients become increasingly unable to care for themselves and perform their usual activities [3]. Thus, HRQoL is a construct that evaluates the impact of physical and mental disorders and disability on an individual’s general well-being and can be measured using generic or disease-specific measures [4].

Caregiver burden is an important multidimensional construct that describes the objective and subjective responses to the physical, psychological, social and financial demands of caring [5]. Caregiver burden has been associated with AD dementia severity: caregivers feel more burdened as AD dementia severity increases [6], although there are some differences between spousal and adult-child caregivers in perceived burden measured using the Zarit Burden Interview (ZBI) [7].

Poorer caregiver HRQoL has not only been associated with increasing patient dependency [3], but also with greater caregiver perceived burden and increased time spent caring [8, 9]. However, two European studies found poor associations between caregiver and patient HRQoL [10, 11] as assessed using the EuroQol-5 dimension questionnaire (EQ-5D), a recognized generic instrument for measuring HRQoL which elicits a utility value (index score) [4]. Patient EQ-5D scores decreased with increasing AD dementia severity, but caregiver EQ-5D scores did not vary by patient disease severity [10, 11]. These findings contradict those from other studies (e.g., [3]) and raise the question of whether caregiver EQ-5D is able to accurately reflect utility/disutility in AD dementia.

Caregiver time spent on instrumental ADL (T-IADL) is an alternative measure of caregiver impact that can be assessed across the full spectrum of disease severity and contributes to informal care costs when cost-of-illness studies take a societal perspective [12]. Time spent on basic ADL such as eating, dressing, and toileting is a less useful measure of caregiver burden in a community-based AD dementia population because basic ADL do not usually become impaired until the moderate-to-severe stages of AD dementia [13]. Caregiver time increases with increasing severity of patient AD dementia [6, 14]

Since the results of studies investigating caregiver HRQoL and burden are inconsistent, further clarification is needed of the role of caregiver EQ-5D scores in assessing the impact of caring for patients with AD dementia and the relationship between EQ-5D and alternative caregiver outcomes. The objective of the present study was to assess the ability of caregiver EQ-5D to measure such impact by exploring EQ-5D index scores and their correlations with ZBI total scores and T-IADL within the caregiver population in the GERAS study. In particular, we used longitudinal data to examine change scores over 18 months and summarized ZBI and T-IADL change scores by EQ-5D domain change category.

## Methods

### Study design and participants

GERAS was an 18-month, prospective, multicenter, naturalistic, observational cohort study conducted in France, Germany, and the UK, designed to evaluate the costs and resource use associated with AD for community-dwelling patients and their caregivers [12]. The study design, patient characteristics, and baseline costs and resource-use data have been reported [12]. The present analysis focuses on the primary caregivers; baseline characteristics and perceived burden have been reported previously for the overall caregiver and patient cohorts [6] and by caregiver relationship to the patient (e.g., adult-child, spouse) [7].

Community-dwelling patients (aged ≥55 years) with probable AD dementia, who presented within the normal course of care and had a Mini-Mental State Examination (MMSE) score of ≤26, were enrolled between October 2010 and September 2011, mostly at specialist secondary care clinics (memory clinics). Patients were also required to have a primary caregiver (responsible for the patient for at least 6 months per year) who was willing to participate in the study.

The study aimed to recruit equal numbers of people in each of three disease severity groups based on MMSE criteria: mild AD dementia (MMSE 21–26 points), moderate AD dementia (MMSE 15–20 points), or moderately severe/severe (MS/S) AD dementia (MMSE ≤14 points). Ethical review board approval was obtained in each country and all participants provided written informed consent before enrollment.

### Data collected

Data were collected for patients and caregivers at the baseline visit and at 6, 12, and 18 months during routine care visits. Full details of the baseline patient and caregiver demographics and characteristics, including comorbidities and medications used, have been reported previously [12].

Caregiver burden was assessed at every visit using the 22-item version of the original 29-item ZBI [15]. The ZBI is a self-report inventory where responses to each item are recorded on a 5-point scale (0 = never to 4 = nearly always) and used to derive a ZBI total score ranging from 0 to 88, with higher scores indicating greater burden. The questions focus on the caregiver’s health, psychological well-being, finances, social life, and relationship with the patient. The ZBI is a valid, reliable, and widely recognized measure of subjective caregiver burden [6, 16].

HRQoL was self-assessed by caregivers at the baseline and 18-month visits using the EQ-5D [17]. Caregivers scored their current health state in each of five domains (pain/discomfort, anxiety/depression, mobility, usual activities, and self-care) using a 3-point scale (1 = no problems, 2 = moderate problems, 3 = extreme problems). From the health-state profile obtained, a scoring algorithm using country-specific preference weights [18] was used to calculate a total utility score (EQ-5D index score) between 0 (represents death) and 1.0 (represents perfect health). Caregivers also rated their current health status on the day of assessment using the EQ-5D visual analog scale (EQ-5D VAS), which ranges from 0 (worst imaginable health) to 100 (best imaginable health).

Caregiver time spent looking after the patient was assessed using the Resource Utilization in Dementia (RUD) instrument [19] (version RUD Complete 3.1), by interview with the caregiver. This is a widely used, standardized instrument for collecting resource-use data in dementia and has been validated for use in different care settings, including in community-dwelling patients [20, 21]. Time (in the month preceding both the baseline visit and the 18-month visit) was recorded as the total number of caregiving hours, including the number of hours spent on basic ADL (e.g., standard self-care tasks such as eating, getting dressed), instrumental ADL (e.g., cooking, shopping), and patient supervision (i.e., watching the patient to prevent dangerous events).

### Statistical analysis

Baseline characteristics of caregivers and patients were summarized based on non-missing observations. Data are presented as means (standard deviations [SDs], medians, and interquartile range [Q1, Q3]) or as numbers and percentages of caregivers/patients.

Comparisons between AD dementia severity groups for caregiver mean EQ-5D, ZBI, and T-IADL used analysis of variance (ANOVA), with country and baseline MMSE severity as independent factors. Although these variables do not follow a normal distribution, the central limit theorem ensures the validity of the ANOVA to compare the means, which are based on a large sample size. Also, because the effect of country was adjusted for in the ANOVA, it allows the combination of country-specific derived EQ-5D index scores.

Spearman correlation coefficients examined the within-subject associations between continuous variables such as caregiver ZBI total scores, EQ-5D index scores, EQ-5D VAS scores, and T-IADL, at baseline, at 18 months, and for the change from baseline to 18 months. We interpreted correlation coefficients of 0–0.19 as very weak and 0.20–0.39 as weak.

The change in caregiver ZBI total score or T-IADL over 18 months was examined for the total population according to the change in each of the five EQ-5D domains, which was categorized as better, stable, or worse based on the numerical changes in each 3-point scale.

All analyses were performed using Statistical Analysis Software (SAS) version 9.2 (SAS Institute, Cary, NC, USA).

## Results

At baseline, the study cohort analyzed comprised 1495 patients with mild AD dementia (*n* = 566), moderate AD dementia (*n* = 472), or MS/S AD dementia (*n* = 457), and their caregivers (*n* = 1495). The number of caregivers and patients from each country was as follows: France, *n* = 419; Germany, *n* = 550; UK, *n* = 526. A total of 1040 caregivers attended the 18-month visit (69.6%). The main reasons for discontinuation from the study were patient institutionalization (*n* = 214), the patient had died (*n* = 92), the caregiver/patient had decided to leave the study (*n* = 119), or the caregiver/patient was lost to follow-up (*n* = 26). The number of caregivers with available data at the 18-month visit was: ZBI, n = 932; EQ-5D, *n* = 934; T-IADL, *n* = 983. The number of caregivers with available data for the change from baseline to 18 months was: ZBI, *n* = 931; EQ-5D, *n* = 933; and T-IADL, *n* = 982.

### Caregiver and patient characteristics

The baseline sociodemographic and clinical characteristics of the caregivers and patients are summarized in Table 1. Caregivers had a mean (SD) age of 67.3 (12.0) years and most were female (64.2%), the spouse of the patient (65.9%), and living with the patient (76.0%). The majority of caregivers reported medical conditions (58.6%; mean of 1.1 medical conditions), most commonly hypertension (36.8%), hypercholesterolemia (23.5%), and depression (10.1%). Approximately one-quarter of the caregivers (23.8%) reported working for pay. The patients had a mean (SD) age of 77.6 (7.7) years, 54.8% were female, mean (SD) time since diagnosis was 2.2 (2.2) years, and 73.6% had comorbidities (mean of 1.4 comorbidities).

**Table 1:** Patient and caregiver characteristics^a^ at baseline

Characteristic	Mean (SD) or n (%)
Caregiver, n	1495
Sex, n (%) female	958 (64.2)
Age, mean (SD)	67.3 (12.0)
Relationship to patient, n (%)	
Spouse	984 (65.9)
Child	405 (27.1)
Other	104 (6.9)
Lives with patient, n (%)	1135 (76.0)
Working for pay, n (%)	355 (23.8)
Caregivers with medical conditions, n (%)	875 (58.6)
Number of medical conditions, mean (SD)	1.1 (1.2)
Patient, n	1495
Sex, n (%) female	819 (54.8)
Age, mean (SD)	77.6 (7.7)
Time since diagnosis of AD, years, mean (SD)	2.2 (2.2)
MMSE score, mean (SD)	17.4 (6.3)
Patients with comorbidities, n (%)	1101 (73.6)
Number of comorbidities, mean (SD)	1.4 (1.2)

A*D* Alzheimer’s disease; *MMSE* Mini-Mental State Examination; *SD* standard deviation

^a^All data are based on patients/caregivers with non-missing data

### Caregiver HRQoL

The caregivers’ overall mean (SD) EQ-5D index score at baseline was 0.84 (0.20), and was 0.86 (0.18), 0.85 (0.19), and 0.82 (0.23) for the mild, moderate, and MS/S AD dementia groups, respectively (ANOVA *P* = 0.043), indicating a very slightly lower caregiver HRQoL for patients with MS/S AD dementia (Table 2). The overall caregiver mean (SD) EQ-5D VAS score at baseline was 75.1 (17.5), and by patient AD severity was 75.8 (16.6), 76.3 (16.5), and 72.9 (19.2) for the mild, moderate, and MS/S AD dementia groups, respectively (ANOVA *P* = 0.013). The mean (SD) change from baseline to 18 months was −0.02 (0.21) for the caregiver EQ-5D index score and −3.0 (18.5) for the caregiver EQ-5D VAS score. For both caregiver EQ-5D measures, the change from baseline to 18 months in the overall sample was not significant and did not differ significantly across the AD dementia severity groups (data not shown).

**Table 2:** Caregiver HRQoL (EQ-5D) and burden scores at baseline

Measure	n	Mean (SD)	Median (Q1, Q3)	*P* value^a^
EQ-5D index score^b^	1483	0.84 (0.20)	0.89 (0.79, 1.00)	
Mild AD dementia	560	0.86 (0.18)	0.89 (0.79, 1.00)	0.043
Moderate AD dementia	469	0.85 (0.19)	0.89 (0.79, 1.00)	
MS/S AD dementia	454	0.82 (0.23)	0.89 (0.73, 1.00)	
EQ-5D VAS score^c^	1483	75.1 (17.5)	80.0 (65.0, 90.0)	
Mild AD dementia	560	75.8 (16.6)	80.0 (69.0, 89.0)	0.013
Moderate AD dementia	469	76.3 (16.5)	80.0 (69.0, 90.0)	
MS/S AD dementia	454	72.9 (19.2)	79.0 (60.0, 89.0)	
ZBI total score^d^	1485	29.0 (15.1)	28.0 (17.0, 40.0)	
Mild AD dementia	560	24.6 (14.2)	22.0 (14.0, 33.0)	<0.001
Moderate AD dementia	471	29.4 (14.8)	29.0 (18.0, 39.0)	
MS/S AD dementia	454	34.1 (14.8)	33.0 (22.0, 45.0)	
T-IADL (hours/month)	1493	79.3 (89.5)	60.0 (20.0, 120.0)	
Mild AD dementia	565	61.0 (83.1)	36.0 (8.0, 90.0)	<0.001
Moderate AD dementia	472	77.5 (79.2)	60.0 (24.0, 120.0)	
MS/S AD dementia	456	103.8 (101.1)	90.0 (40.0, 120.8)	

*AD* Alzheimer’s disease; *EQ-5D* EuroQol-5 dimension questionnaire; *HRQoL* health-related quality of life; *MS/S* moderately severe/severe; *Q1, Q3* interquartile range; *SD* standard deviation; *T-IADL* time spent on instrumental activities of daily living; *VAS* visual analog scale; *ZBI* Zarit Burden Interview

Missing data (overall): EQ-5D, *n* = 12; ZBI, *n* = 10; T-IADL, *n* = 2

^a^Comparisons between AD severity groups used analysis of variance (ANOVA) with country and baseline Mini-Mental State Examination (MMSE) severity as independent factors

^b^Country-specific health status index score; range 0 to 1.0, higher scores indicate better health-related quality of life

^c^EQ-5D VAS score range = 0–100, higher scores indicate better health-related quality of life

^d^ZBI total score range = 0–88, higher scores indicate greater burden

**Fig. 1:**
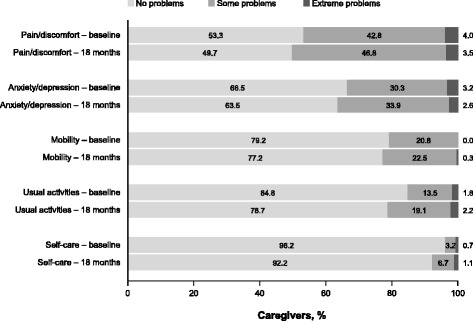
Problems in caregiver EQ-5D domains at baseline and 18 months. Baseline: *n* = 1483 (data missing for 12 caregivers); 18 months: *n* = 934 (data missing for 113 caregivers). EQ-5D, EuroQol-5 dimension questionnaire

Examination of the EQ-5D domain scores (Fig. 1) showed that very few caregivers had extreme problems (<5% for any domain) and the levels of problems were similar at baseline and at 18 months. At both time points, more caregivers had some problems in the domains of pain/discomfort (baseline: 42.8%; 18 months: 46.8%) and anxiety/depression (baseline: 30.3%; 18 months: 33.9%) than in the other three domains.

### Caregiver burden

ZBI total scores at baseline ranged from 0 to 80 and the mean (SD) score was 29.0 (15.1). The mean (SD) ZBI total score showed a greater burden for the patient groups with worse disease severity: 24.6 (14.2), 29.4 (14.8), and 34.1 (14.8) for the mild, moderate, and MS/S AD dementia groups, respectively (ANOVA *P* < 0.001) (Table 2). The mean (SD) change in ZBI total score from baseline to 18 months was 4.9 (12.6) for the overall cohort of caregivers, and differed significantly according to patient disease severity at baseline: 5.4 (11.8), 5.9 (13.7), and 3.0 (12.4) for the mild, moderate, and MS/S AD dementia groups, respectively (ANOVA *P* = 0.028).

### Caregiver T-IADL

Median caregiver T-IADL during the month before baseline was 60.0 (Q1, Q3: 20.0, 120.0) hours (Table 2). This time increased for the patient groups with greater disease severity and was 36.0 (Q1, Q3: 8.0, 90.0), 60.0 (Q1, Q3: 24.0, 120.0), and 90.0 (Q1, Q3: 40.0, 120.8) hours for the month before baseline in the mild, moderate, and MS/S AD dementia groups, respectively (ANOVA *P* < 0.001; see Table 2). The median change from baseline to 18 months in T-IADL was 10.0 (Q1, Q3: −15.0, 45.0) hours/month for the overall cohort; the median changes from baseline in the mild, moderate, and MS/S AD dementia groups were 13.0 (Q1, Q3: −5.0, 52.0), 10.0 (Q1, Q3: −15.0, 46.5), and 0.0 (Q1, Q3: −30.0, 45.0) hours/month, respectively (ANOVA *P* = 0.151).

### Correlations Between EQ-5D, ZBI, and T-IADL

Correlations were weak or very weak between EQ-5D (index and VAS scores) and ZBI or T-IADL at each time point and for the change scores (Table 3). For the ZBI, the 18-month change score correlation was −0.16 (95% confidence interval, CI: −0.22, −0.09) for the EQ-5D VAS score and −0.09 (95% CI: −0.15, −0.03) for the EQ-5D index score. Caregiver T-IADL showed weak correlation with ZBI scores, and the 18-month change score correlation was 0.12 (95% CI: 0.05, 0.18). However, there was no significant correlation between the 18-month change scores for T-IADL and the EQ-5D index score (Table 3).

**Table 3:** Correlations^a^ between caregiver EQ-5D, T-IADL, and ZBI scores

Variable	Time point	EQ-5D VAS score				EQ-5D index score				T-IADL			
		n	Coefficient	95% CI	*P* value	n	Coefficient	95% CI	*P* value	n	Coefficient	95% CI	*P* value
ZBI total score	Baseline vs baseline	1483	−0.21	−0.26, −0.16	<0.001	1483	−0.21	−0.25, −0.16	<0.001	1484	0.30	0.25, 0.34	<0.001
	18 months vs 18 months	929	−0.19	−0.26, −0.13	<0.001	929	−0.21	−0.27, −0.15	<0.001	928	0.22	0.15, 0.28	<0.001
	Change score vs change score^b^	928	−0.16	−0.22, −0.09	<0.001	928	−0.09	−0.15, −0.03	0.006	926	0.12	0.05, 0.18	<0.001
T-IADL	Baseline vs baseline	1482	−0.15	−0.19, −0.10	<0.001	1482	−0.14	−0.19, −0.09	<0.001				
	18 months vs 18 months	930	−0.08	−0.15, −0.02	0.010	930	−0.10	−0.16, −0.03	0.004				
	Change score vs change score^b^	928	−0.07	−0.13, −0.01	0.030	928	0.02	−0.04, 0.08	0.546				

*CI* confidence interval; *EQ-5D* EuroQol-5 dimension questionnaire; *T-IADL* time spent on instrumental activities of daily living (hours/month); *VAS* visual analog scale; *ZBI* Zarit Burden Interview

^a^Spearman correlation coefficients, 95% CIs, and *P* values

^b^Change in score from baseline to 18 months

### ZBI Scores by EQ-5D Domain

**Fig. 2:**
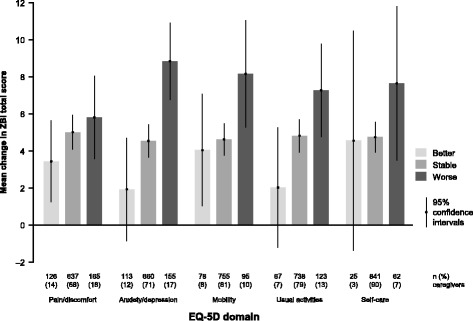
Change in caregiver ZBI total score by EQ-5D domain change category from baseline to 18 months (*n* = 931). Change in EQ-5D domain data were missing for three of these caregivers. EQ-5D, EuroQol-5 dimension questionnaire; ZBI, Zarit Burden Interview

The mean ZBI total scores at baseline and at 18 months by EQ-5D domain showed that burden tended to be greater (higher mean ZBI total score) among caregivers with greater problem severity in each EQ-5D domain (data not shown). From baseline to 18 months, the HRQoL of the majority of caregivers (68–90%) was stable in each EQ-5D domain, though some caregivers (3–14%) showed better EQ-5D scores in some domains than at baseline. HRQoL worsened from baseline to 18 months in 7% of caregivers within the self-care domain, 10% within the mobility domain, 13% in the usual activities domain, 17% in anxiety/depression domain, and 18% in the pain/discomfort domain (Fig. 2).

As evidenced by Fig. 2, which shows the mean change in ZBI total score by EQ-5D domain change category over 18 months, there was a trend for caregivers who experienced a worsening in EQ-5D domain score over 18 months to have the largest increases in ZBI total score (i.e., the greatest increase in caregiver burden).

### T-IADL by EQ-5D Domain

**Fig. 3:**
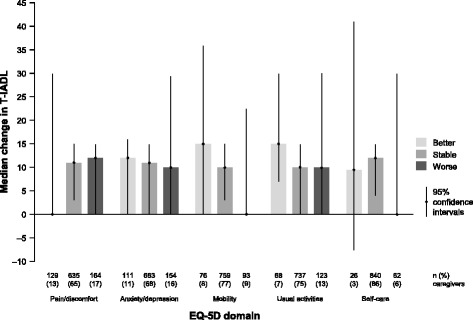
Change in caregiver T-IADL by EQ-5D domain change category from baseline to 18 months (*n* = 982). Change in EQ-5D domain data were missing for 54 of these caregivers. 95% confidence intervals are bootstrap-based. EQ-5D, EuroQol-5 dimension questionnaire; T-IADL, time spent on instrumental activities of daily living (hours/month)

Figure 3 summarizes the median change in caregiver T-IADL by EQ-5D domain change category over 18 months. Of the 982 caregivers with available data on the change in T-IADL, the EQ-5D domain change category was missing for 54 (5%). Fig. 3 shows that there was no clear pattern for median change in T-IADL by EQ-5D domain change category.

## Discussion

The impact of AD dementia on caregivers is substantial and should be considered when evaluating the societal impact of this disease. Although caregiver outcomes are not typically included in economic evaluations, a preference-based measure such as the EQ-5D can be used to inform economic evaluations [7, 22]. However, the results of our analysis suggest that the EQ-5D is not particularly effective for capturing the true impact on caregivers of caring for people with AD dementia: the EQ-5D index score had a low sensitivity to change over an 18-month period and was not clearly differentiated by patient AD dementia severity. EQ-5D index and VAS scores had weak or very weak correlations with other potential measures of impact, perceived caregiver burden (ZBI) and a measure of caregiver time (caregiver T-IADL), both assessed using standardized instruments.

When the EQ-5D was analyzed at the domain level, caregivers who experienced a worsening in HRQoL domain score over 18 months had a greater increase in mean ZBI total score than caregivers whose HRQoL domain score remained stable or improved. However, this trend was not observed when the EQ-5D domains were combined into the single EQ-5D index score (Table 3 shows a very weak correlation between the change scores).

The longitudinal GERAS study included a large sample of community-dwelling patients with a wide range of AD dementia severity who participated with their caregivers, with assessments being made in a naturalistic setting. The caregivers participating in the GERAS study had a high mean EQ-5D index score (0.84) at baseline, showing that they had relatively good HRQoL, similar to that of the general population of people aged 65–74 years in each country (France: 0.81; Germany: 0.89; and UK: 0.78 from Table 3.6 in Szende et al. [18]). The EQ-5D index score from our study is also consistent with a previous study showing little difference in utility values in a UK general population sample (*n* = 77) and caregivers of people with mild dementia (*n* = 71), where the mean (SD) EQ-5D scores were 0.79 (0.25) for the general population sample and 0.78 (0.19) for the caregivers [23]. However, these caregivers of people with dementia gave lower utility values for dementia health states than members of the general population; this could impact on the results of cost–utility analyses, which generally use general population values [23].

There were minimal changes in caregiver EQ-5D scores over the 18-month follow-up period. However, as the EQ-5D is a brief generic measure of health status rather than an AD-specific HRQoL scale, it may miss both disease-specific and caregiver-specific impacts [4]. The majority of caregivers reported no problems in the EQ-5D domains of mobility, self-care, and usual activities, which reflect physical health, implying that these caregivers generally had the good physical health to be able to perform their role as caregiver. Approximately half of the caregivers reported some or extreme problems in the pain/discomfort domain and about one-third of caregivers had some or extreme problems in the anxiety/depression domain, reflecting emotional/mental health. This is consistent with previous reports that a high proportion of informal caregivers of patients with AD dementia report problems in the EQ-5D domains of pain/discomfort and anxiety/depression [3, 9]. As the GERAS study required caregivers to be the established caregiver, the impact on pain/discomfort and anxiety/depression scores may have been captured at baseline with low caregiver expectation of much change over time. These findings suggest that, because some of the EQ-5D domains are at ceiling scores (with few caregivers remaining in the study expected to decline much in the physical or emotional domains), caregiver EQ-5D is not a particularly informative or sensitive measure of the impact of caring for patients with AD dementia. It is possible that most caregivers who remained in the study were physically healthy and that patients discontinuing the study (for reasons including institutionalization or death) were those experiencing more acute problems. The discontinuation of caregivers of these patients may thereby reduce the likelihood of observing caregivers with extreme problems.

In our study, the increase in ZBI total score over time differed according to patient AD dementia severity at baseline and was highest for those caring for patients with moderate AD dementia and lowest for caregivers of patients with MS/S AD dementia. The greatest change in caregiver burden may be expected to occur in moderate AD dementia because patients often develop more behavioral problems during this stage of the disease; we believe that by the time the patient has progressed to more severe AD dementia, caregivers are likely to have adapted their life and reached a peak in their perceived burden, and patients may be receiving more formal care. There was a tendency for the increase in mean ZBI total score to be greatest among caregivers who showed a worsening in each of the EQ-5D domains, especially the anxiety/depression domain.

Little published information exists on the relationship between caregiver burden and HRQoL over time in dementia. However, our finding that EQ-5D is a relatively insensitive measure of the impact of caring is consistent with a previous longitudinal study where the mean ZBI score was higher for caregivers of home-living patients with dementia (32.4) than for caregivers of patients with dementia in long-term care (24.9), while the mean EQ-5D index score was similar for both types of caregiver (0.76 and 0.78, respectively) [24]. Furthermore, for a subgroup of caregivers where the patient transitioned from home to institutional care during the 3-month follow-up period, the mean ZBI score decreased from 35.4 to 22.4, while the caregiver EQ-5D index score remained stable at 0.77 [24].

Caregiver T-IADL is sensitive to change across the full range of AD dementia severity and is a common outcome measure in clinical trials. Our analyses showed that caregiver T-IADL had very weak correlations with caregiver EQ-5D scores (index and VAS scores), and that there was no clear pattern for the change in caregiver T-IADL by change in EQ-5D domains, reinforcing our conclusion that EQ-5D is not a sensitive measure of the impact of caring for people with AD dementia.

The relationship between caregiver HRQoL and T-IADL needs further investigation. A recent systematic review on the relationship between caregiver quality of life (QoL, covering the broader concept of complete physical, mental, and social well-being) and the level/quality of care provided to people with AD included only one study, QoL was measured as overall well-being using the Perceived Change Index, and the evidence was equivocal [25]. An increase in caregiver QoL over six months was associated with an increase in T-IADL, although there was a decrease in total caregiving hours [25]. This suggests that caregivers with a better HRQoL are able to spend more time addressing specific aspects of care related to instrumental ADL, such as housework, financial management, and correct use of medications. Thus, we can speculate that interventions which improve caregiver HRQoL may improve the level of care that caregivers provide to people with AD dementia.

Taken together, our results imply that the EQ-5D may not be the most appropriate measure of the impact of caring for people with AD dementia given the structure of its domains. This suggests that, although the EQ-5D is commonly used in health economics and outcomes research involving caregivers of people with dementia [26], it may not be sensitive enough to measure changes in caregiver HRQoL. Thus, the EQ-5D may underestimate the impact of an intervention on the caregiver and, therefore, its cost-effectiveness.

Other instruments have been developed that measure the impact of caregiving on informal caregivers, which can be used in economic evaluations; these include the Care-related Quality of Life instrument (CarerQoL) [27, 28] and the Caregiver Experience Scale (CES) [29, 30]. However, these instruments are still undergoing validation.

There are several limitations to our study. First, the GERAS study included only community-dwelling patients at enrollment who had primary caregivers willing to participate in the study. Thus, our sample of caregivers may not be representative of all caregivers for AD, as it includes only those able/willing to participate. This is reflected in the high proportion of caregivers who had no problems in the EQ-5D domains of mobility, self-care, and usual activities, although we were able to examine changes in EQ-5D over time. Second, as HRQoL (measured using EQ-5D) is a different construct from caregiver burden and time spent on ADL, this has imposed some limitation on our analysis. However, our aim was to understand how these commonly reported measures improve our understanding of the impact of AD for the caregiver and enhance understanding of how the measures best align with the decline in patients’ AD severity. Third, other factors that may influence caregiver HRQoL, burden and time spent caring, such as depression in the caregiver or neuropsychiatric symptoms in the person with AD dementia [2, 6, 31, 32], were not included within the current objective of our analyses. Fourth, our analysis is based only on those caregivers with available data at 18 months, and evaluated score data at baseline and 18 months only. In addition, we used pooled data from three countries and it would be interesting to examine whether there are country-level effects. As part of our descriptive summary of longitudinal GERAS study results, we investigated the EQ-5D index scores from all countries based on the UK population values and have seen little difference from those generated using country-specific values (data available on request). We found no evidence to suggest that the relationship between EQ-5D and the other measures would differ by country. Fifth, changes in ZBI and T-IADL cannot be compared in the same way as EQ-5D domain changes, as they are continuous scores. Assessment of T-IADL by the caregiver may be subject to recall bias although an electronic diary to record time spent giving care was provided for the purposes of this study. Finally, caregiver HRQoL and burden may change after the patient with AD dementia has been institutionalized [33, 34]. We will report caregiver EQ-5D and ZBI after patient institutionalization or death in a future publication.

## Conclusions

Our findings indicate that EQ-5D may not be the best measure of the impact on caregivers of caring for people with AD dementia because it mainly focuses on physical health. Alternative measures, including measures of caregiver burden assessed using the ZBI or caregiver time, may provide a more accurate picture of the impact of caring for a person with AD dementia, and require further investigation. This approach of assessing caregiver burden is perhaps more relevant than looking at HRQoL, as the current emphasis is on developing interventions aimed at relieving caregiver burden.

## Abbreviations

AD: Alzheimer’s disease
ADL: Activities of daily living
ANOVA: Analysis of variance
CarerQoL: Care-related Quality of Life instrument
CES: Caregiver Experience Scale
EQ-5D: EuroQol-5 dimension questionnaire
HRQoL: Health-related quality of life
MMSE: Mini-Mental State Examination
MS/S: Moderately severe/severe
Q1, Q3: Interquartile range
QoL: Quality of life
RUD: Resource Utilization in Dementia
SAS: Statistical Analysis Software
SD: Standard deviations
T-IADL: Time spent on instrumental ADL
VAS: Visual analog scale
ZBI: Zarit Burden Interview
